# Structure of centromere chromatin: from nucleosome to chromosomal architecture

**DOI:** 10.1007/s00412-016-0620-7

**Published:** 2016-11-17

**Authors:** Thomas Schalch, Florian A. Steiner

**Affiliations:** Department of Molecular Biology, Sciences III, Institute of Genetics and Genomics of Geneva (iGE3), University of Geneva, Geneva, Switzerland

**Keywords:** Centromere, CENP-A, Pericentromere, Cohesin, Chromatin

## Abstract

The centromere is essential for the segregation of chromosomes, as it serves as attachment site for microtubules to mediate chromosome segregation during mitosis and meiosis. In most organisms, the centromere is restricted to one chromosomal region that appears as primary constriction on the condensed chromosome and is partitioned into two chromatin domains: The centromere core is characterized by the centromere-specific histone H3 variant CENP-A (also called cenH3) and is required for specifying the centromere and for building the kinetochore complex during mitosis. This core region is generally flanked by pericentric heterochromatin, characterized by nucleosomes containing H3 methylated on lysine 9 (H3K9me) that are bound by heterochromatin proteins. During mitosis, these two domains together form a three-dimensional structure that exposes CENP-A-containing chromatin to the surface for interaction with the kinetochore and microtubules. At the same time, this structure supports the tension generated during the segregation of sister chromatids to opposite poles. In this review, we discuss recent insight into the characteristics of the centromere, from the specialized chromatin structures at the centromere core and the pericentromere to the three-dimensional organization of these regions that make up the functional centromere.

## Introduction

The centromere is a conserved and essential feature of eukaryotic chromosomes that enables the equal segregation of genetic material into daughter cells during cell division. The centromere generally appears as primary constriction of mitotic chromosomes, first described by Walther Flemming in 1882 (Flemming 1882). It was apparent early on that chromosomes are segregated by attachment of microtubules to the primary constriction. The kinetochore—the proteinaceous structure that links the chromosomes to the microtubules for the segregation of chromosomes—was described in the 1960s (Luykx 1965; Brinkley and Stubblefield 1966; Jokelainen 1967) and has since been studied in great detail. The structure and organization of the chromatin forming the centromere, however, have remained more obscure. One key feature of centromeric chromatin is the incorporation of the histone H3 variant centromere protein A (CENP-A), also called cenH3 (Earnshaw and Rothfield 1985; Palmer et al. 1987; Talbert et al. 2012; Earnshaw et al. 2013; Talbert and Henikoff 2013). CENP-A is an almost universally conserved centromeric protein that plays a key role in specifying the centromere, where it replaces H3 in centromeric nucleosomes and is necessary for centromere function (McKinley and Cheeseman 2016). As is the case for CENP-A, many protein components of centromeric chromatin are conserved, but the organization of CENP-A nucleosomes and the DNA sequences on which the centromere is established vary widely between taxa (Steiner and Henikoff 2015). DNA at the centromere is often characterized by tandemly repeated sequence. Tandem repeat monomer lengths vary between species but in many cases roughly correspond to nucleosomal units (Melters et al. 2013). These repeats, however, are not required for centromere function, as neocentromeres can form in virtually any sequence context (Scott and Sullivan 2014). Despite the divergence in DNA sequence and CENP-A organization, centromeric chromatin can in most organisms be separated into two regions: the centromere core, marked by CENP-A nucleosomes, and a pericentromeric region, usually associated with heterochromatin. Functional studies have shown that disruption of the specialized chromatin in both regions leads to defects in chromosome segregation, but the mechanistic details of the interplay between the two regions are not well understood. Here, we summarize recent progress in understanding how the centromere core and the pericentric region work together in establishing a three-dimensional structure that enables attachment to microtubules via kinetochore complex and that supports the tension generated during the segregation of chromosomes.

### Characterization of the centromere core

Centromere cores are defined by the presence of CENP-A. The number of CENP-A nucleosomes varies between species from a single one that occupies ∼120 bp sequence in *Saccharomyces cerevisiae* to several hundred that are embedded in megabases of satellite DNA in humans (Pluta et al. 1995; Furuyama and Biggins 2007; Aldrup-Macdonald and Sullivan 2014; Bodor et al. 2014). Together with additional centromeric chromatin components, CENP-A nucleosomes create a permissive environment for centromere establishment and maintenance and are involved in the formation of three-dimensional structures that link to kinetochores and serve as capture devices for the mitotic microtubules. Recent research has advanced our understanding of how CENP-A nucleosomes are able to define the centromere core and revealed new features of the centromere core chromatin that are essential for centromere function.

### CENP-A residues that specify the centromere

CENP-A is conserved among virtually all eukaryotic lineages and specifically marks the centromere. However, in contrast to other histone variants, there is little or no amino acid sequence conservation outside the C-terminal histone fold domain, and the N-terminal tails vary widely both in size and sequence (Malik and Henikoff 2003). Despite this divergence, *S. cerevisiae* CENP-A can complement human CENP-A, and distant plant CENP-As can complement *Arabidopsis thaliana* CENP-A, indicating that the functional features remain conserved (Wieland et al. 2004; Ravi et al. 2010; Maheshwari et al. 2015).

**Fig. 1:**
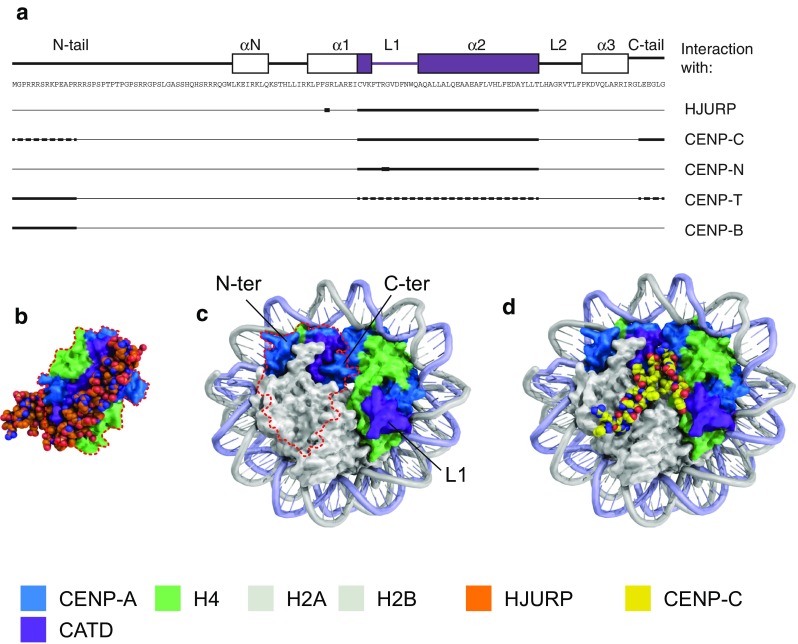
CENP-A residues that specify the centromere. **a** Primary sequence and schematic representation of the secondary structure of human CENP-A (*top*). The CATD is highlighted in *purple*. Regions of the CENP-A protein that interact with HJURP and different CCAN components (*bottom*). **b** Structural model of the CENP-A/H4 heterodimer in complex with HJURP (PDB ID 3R45) (Hu et al. 2011). **c** Structural model of the CENP-A nucleosome (PDB ID 3AN2) (Tachiwana et al. 2011). The CENP-A/H4 heterodimer on the *left* is shown in the same orientation as in **b** (framed by *red dotted line*), illustrating how HJURP binding prevents formation of a (CENP-A/H4)_2_ tetramer and association with DNA. **d** Structural model of CENP-C bound to the CENP-A nucleosome (CENP-C from PDB ID 4X23 modeled on CENP-A nucleosome from PDB ID 3AN2) (Tachiwana et al. 2011; Kato et al. 2013)

The in vivo structure and composition of CENP-A nucleosomes remain a controversial area of research that has been extensively covered in other reviews, e.g., Quénet and Dalal (2012), Henikoff and Furuyama (2012), Kurumizaka et al. (2013), Padeganeh et al. (2013), Fukagawa and Earnshaw (2014), and Steiner and Henikoff (2015). In vitro, human CENP-A is incorporated into an octameric nucleosome that is largely superimposable with the H3 nucleosome (Tachiwana et al. 2011). Comparison of the crystal structure of a human CENP-A-containing octamer with that of the H3-containing octamer revealed two major differences in the histone fold domain: CENP-A loop 1 protrudes from the CENP-A nucleosome and exposes two extra amino acid residues (Arg 80 and Gly 81), and the αN helix of CENP-A is about one turn shorter than in H3, which affects how DNA is wrapped around the CENP-A-containing nucleosome (Tachiwana et al. 2011; Roulland et al. 2016). These findings are in line with experiments using chimeras of H3 and CENP-A that identified a region covering loop 1 and α2 helix, termed CENP-A targeting domain (CATD), as the main CENP-A domain to drive centromere localization and function (Black et al. 2004; Black et al. 2007). A series of recent papers have refined our understanding of how the CATD and additional residues of CENP-A interact with other centromeric proteins to mediate centromere establishment and function (Fig. 1a).

Human CENP-A interacts with its histone chaperone HJURP via CATD, and this interaction is essential for targeting CENP-A to the centromere (Foltz et al. 2009; Fachinetti et al. 2015). Crystal structures of human and *S. cerevisiae* HJURP/CENP-A/H4 heterotrimers revealed that the CATD interacts with the N-terminal part of HJURP, while the C-terminal domain of HJURP caps the DNA-binding region of the CENP-A/H4 heterodimer, preventing formation of a (CENP-A/H4)_2_ tetramer and premature association with DNA (Zhou et al. 2011; Hu et al. 2011; Cho and Harrison 2011) (Fig. 1b). Six surface-exposed human CENP-A CATD residues (four in *S. cerevisiae* CENP-A) are the main drivers of interaction, while binding of HJURP at the α1 helix restricts the conformational flexibility of the CENP-A/H4 dimer (Zhou et al. 2011; Bassett et al. 2012; Zhao et al. 2016). In addition to the CATD, interaction with HJURP also involves serine 68 in the α1 helix (Hu et al. 2011; Logsdon et al. 2015), which needs to be dephosphorylated for stable interaction (Yu et al. 2015). These findings explain how CENP-A is discriminated from H3 for targeted incorporation at the centromere. Since interaction of HJURP with CENP-A is incompatible with DNA binding, HJURP has to dissociate from the CENP-A/H4 dimer for incorporation into the nucleosome, thereby making the CATD available for interaction with other factors (Fig. 1c). In most organisms, CENP-A serves as an epigenetic mark for the inheritance of centromeres during mitosis and meiosis, and new CENP-A nucleosomes are recruited to sites of pre-existing CENP-A. For recent reviews on the regulation of CENP-A loading, refer to Nechemia-Arbely et al. (2012), Stellfox et al. (2013), Müller and Almouzni (2014), McKinley and Cheeseman (2016), Chen and Mellone (2016), and Nagpal and Fukagawa (2016).

**Fig. 2:**
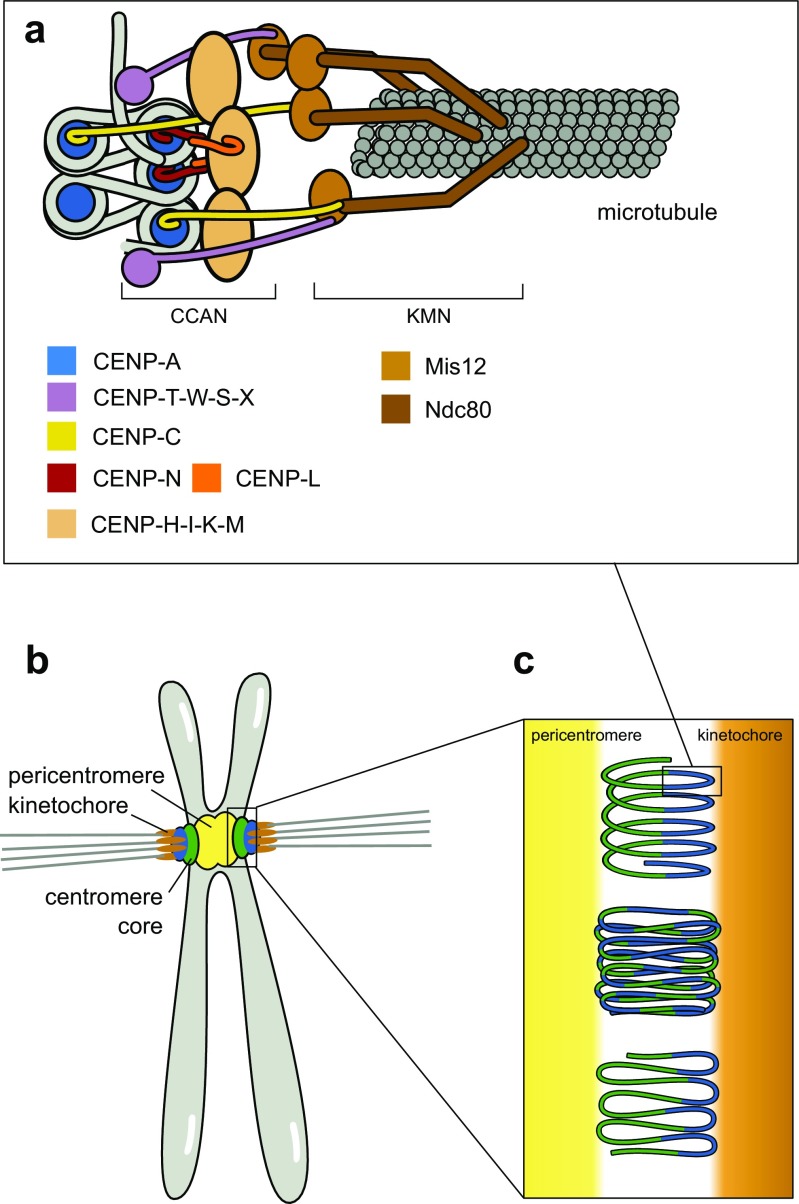
Three-dimensional arrangement of the centromere during mitosis. **a** Cartoon of kinetochore complex linking CENP-A chromatin to the microtubules. CENP-A nucleosomes coordinate the protein network of the CCAN, which recruits the outer kinetochore (KMN network) that attaches to the microtubules. **b** The pericentromere provides cohesion between sister chromatids and acts as a foundation for the centromere core, which assembles the kinetochore complexes for the attachment of microtubules. **c** Chromatin of the centromere core is folded to expose the CENP-A nucleosomes to the surface of the primary constriction. Several models for this assembly have been proposed: solenoid model (*top*) (Blower et al. 2002), layered boustrophedon (*middle*) (Ribeiro et al. 2010), and looping model (*bottom*) (Blower et al. 2002)

CENP-A specifies chromatin at the centromere core, but centromere function also requires a set of proteins that form the constitutive centromere-associated network (CCAN). Once deposited, CENP-A interacts with CENP-B and a number of CCAN components, including CENP-C, CENP-N, and CENP-T, and these, in turn, interact with other CCAN components, forming a network of inner kinetochore proteins (Figs. 1 and 2a).

Of the CCAN components, CENP-C is most closely associated with the CENP-A nucleosome. Human CENP-C also possesses a DNA-binding motif (Sugimoto et al. 1994), but it is unclear if it binds DNA independently of the CENP-A nucleosomes. Using H3/CENP-A chimeras, it was shown that CENP-C recognizes the C-terminal part of CENP-A in humans and that just the C-terminal six amino acids of CENP-A are sufficient for CENP-C recruitment and kinetochore assembly in *Xenopus laevis* egg extracts (Carroll et al. 2010; Guse et al. 2011). This suggested that the CATD is mainly required for CENP-A targeting, but once it is deposited, the kinetochore is recruited via C-terminal domain and CENP-C. However, follow-up *Xenopus* studies showed that the CATD also has a role in CENP-C recruitment, especially at lower densities of CENP-A nucleosomes (Westhorpe et al. 2015). Experiments targeting human CENP-A/H3 chimeras to ectopic loci confirmed the need for both the C-terminus and the CATD for CENP-C recruitment and uncovered that residues within the N-terminus further enhance this interaction (Logsdon et al. 2015). Although CENP-C homologs have been identified in all model organisms, not all parts of the protein are fully conserved, and the way it binds to the CENP-A nucleosome differs between species. The central region of CENP-C is critical for this interaction in mammals, while in chicken cells, only the C-terminal part of CENP-C is required (Carroll et al. 2010; Kato et al. 2013; Nagpal et al. 2015). By NMR and crystal structure, the CENP-C central domain was shown to bind to the nucleosome surface by docking to the acidic patch (Kato et al. 2013) (Fig. 1d). These results explain the low conservation but universally hydrophobic nature of CENP-A C-termini (in contrast to H3 C-termini) across species. Binding of human CENP-C flattens and rigidifies CENP-A nucleosomes and limits the rate at which they turn over at centromeres (Falk et al. 2015; Falk et al. 2016). CENP-C interacts with CENP-H-K-I-M and CENP-L-N complexes via its central domain and is important for bridging the centromeric chromatin to the outer kinetochore, as shown in human and chicken cells (Klare et al. 2015; Nagpal et al. 2015; McKinley et al. 2015) (Fig. 2a).

The CATD of human CENP-A is also required for interaction with CENP-N, which is independent of CENP-C recruitment (Carroll et al. 2009; Logsdon et al. 2015; Fang et al. 2015). This suggests that there may be competition between CENP-C and CENP-N for binding at the CENP-A CATD, but the significance of this competition is unclear. The exposed RG loop of CENP-A plays a key role in CENP-N recruitment (Fang et al. 2015). The RG loop is concealed by centromeric chromatin compaction, and access to the RG loop by CENP-N requires decompaction of CENP-A-containing chromatin fiber, which occurs mainly at S phase.

The interaction of human CENP-A with CENP-T is mediated by a combination of binding to the N-terminal and C-terminal regions and the CATD but requires the presence of CENP-C and CENP-N and may therefore be indirect (Logsdon et al. 2015; Tachiwana et al. 2015). In *Schizosaccharomyces pombe*, the CENP-A N-terminus seems to be the major driver for the interaction with CENP-T (Folco et al. 2015). CENP-T particles have been mapped to the linker regions between CENP-A nucleosomes at both *S. pombe* and human centromeres (Thakur et al. 2015; Thakur et al. 2016).

Removal of ectopic CENP-A deposited outside of the centromere is best studied in *S. cerevisiae*. The E3 ubiquitin ligase Psh1 mediates degradation of ectopic CENP-A that otherwise accumulates at euchromatic loci such as promoters and can lead to segregation defects (Hewawasam et al. 2010; Ranjitkar et al. 2010; Hildebrand and Biggins 2016). Interestingly, targeting of CENP-A by Psh1 depends on the CATD, adding another function to this region of the protein. As a consequence, nucleosomal CENP-A is a poor substrate for Psh1 and requires the facilitates chromatin transcription (FACT) complex for efficient targeting (Choi et al. 2012; Deyter and Biggins 2014). FACT thus can play an important role in chromosome segregation both by ensuring the degradation of ectopically incorporated CENP-A and by facilitating the deposition of CENP-A at the centromere core (discussed below) (Chen et al. 2015; Okada et al. 2009). CENP-A ubiquitination is antagonized by the SAGA complex, and the right balance between the two activities is important, as the mitotic instabilities observed in SAGA mutants can be rescued by Psh1 deletion (Canzonetta et al. 2015).

### CENP-T, CENP-W, CENP-S, CENP-X, and CENP-B contribute to centromeric chromatin

Centromeric chromatin is generally defined by the presence of CENP-A nucleosomes. However, other centromere-specific proteins may help to shape the chromatin at the centromere by binding centromeric DNA independently of CENP-A. The CCAN components CENP-T, CENP-W, CENP-S, and CENP-X have been identified independently, CENP-T and CENP-S as components of CENP-A chromatin (Foltz et al. 2006; Izuta et al. 2006), CENP-X as a subcomplex component with CENP-S (Foltz et al. 2006; Amano et al. 2009), and CENP-W as an interactor with CENP-T (Hori et al. 2008). Crystal structures revealed that CENP-T, CENP-W, CENP-S, and CENP-X are histone fold proteins that heterotetramerize, bind to, and supercoil DNA (Nishino et al. 2012). The resulting particle wraps about 100 bp of DNA, presumably in a single wrap. Both heterotetramerization and DNA binding are required for the assembly of a functional kinetochore. Interestingly, the CENP-T-W-S-X complex induces positive supercoils (Takeuchi et al. 2014), as has been shown for *S. cerevisiae* point centromeres (Furuyama and Henikoff 2009; Díaz-Ingelmo et al. 2015). CENP-T, CENP-W, CENP-S, and CENP-X are essential for centromere function, and CENP-T can recruit a functional centromere when tethered to DNA (Gascoigne et al. 2011; Nishino et al. 2012; Hori et al. 2013). They may thus provide an alternative mechanism to link the kinetochore to chromatin that partially bypasses CENP-A nucleosomes. How this works on a molecular level remains to be addressed, especially given that some lineages lack some or all of these proteins. While tethering of CENP-T recruits only outer kinetochore components, tethering of CENP-C, CENP-I, or HJURP recruits CENP-A and establishes a fully functional CCAN in chicken cells (Hori et al. 2013) and highlights the interdependence of CENP-A deposition and CCAN formation.

As mentioned above, DNA sequences that underlie the centromeres vary greatly between organisms, while the centromere-specific proteins are relatively well conserved. As a consequence, most centromeric proteins do not show affinity for a specific sequence. The best-known exception to this rule is CENP-B, which specifically binds to a conserved DNA sequence called CENP-B box within the satellite repeats at centromeres (Masumoto et al. 1989; Muro et al. 1992). CENP-B has been independently domesticated from pogo-like transposase in several lineages including mammals, *S. pombe*, and insects (Mateo and González 2014). CENP-B interacts with the N-terminal tail of CENP-A (Fachinetti et al. 2013), and its binding to CENP-B boxes in the vicinity of CENP-A stabilizes the CENP-A nucleosome on alphoid DNA (Fujita et al. 2015). Based on MNase digestion patterns, CENP-B separates two tandem CENP-A nucleosome on human alpha-satellite arrays in vivo (Henikoff et al. 2015). The functional importance of the CENP-B box and CENP-B itself is not entirely understood. There are examples of centromeres without CENP-B boxes, e.g., on the human Y-chromosome, at neocentromeres, or at all centromeres in some primates (Masumoto et al. 1989; Voullaire et al. 1993; Haaf et al. 1995; Goldberg et al. 1996), and the CENP-B protein is not essential in mice (Kapoor et al. 1998; Hudson et al. 1998; Perez-Castro et al. 1998). Moreover, *S. pombe* CENP-B homologs play a role in host genome surveillance for retrotransposons and replication fork progression through transposons but have no significant role in chromosome segregation (Cam et al. 2008; Zaratiegui et al. 2011). However, a recent study has shown that human CENP-B binds to and stabilizes CENP-A and CENP-C and enhances the fidelity of centromere function (Fachinetti et al. 2015). Moreover, CENP-B boxes are required for the formation of functional human artificial chromosomes (Ohzeki et al. 2002). Therefore, CENP-B may not be strictly required for centromere function but may contribute to the stability and maintenance of centromeres.

### “Active” chromatin and transcription at centromere cores

Post-translational histone modifications correlate with domains of chromatin organization and can influence how chromatin is packaged. Histone marks that correlate with transcriptional activity tend to be associated with less tightly packaged chromatin than histone marks that correlate with transcriptional inactivity. Chromatin at the centromere core is characterized by H3K4me2, generally associated with active chromatin, that is interspersed with CENP-A. This contrasts with the predominant H3K9me3 found at pericentric chromatin (Sullivan and Karpen 2004; Lam et al. 2006). Centromeric H3K4me2 is functionally important, as it is required for HJURP targeting and CENP-A assembly on a synthetic human kinetochore (Bergmann et al. 2012). H2B monoubiquitination, another modification associated with actively transcribed chromatin, is needed for centromere integrity, as H2Bub depletion leads to heterochromatization of centromere cores and deficient chromosome segregation (Sadeghi et al. 2014). However, the centromere core is not homogeneously covered, and H3-rich domains associated with high H3K9me3 and low H3K4me2 levels are also observed at the centromere core (Ribeiro et al. 2010). The balance between modifications associated with active or silent chromatin seems to be important: At human artificial chromosomes, altering the chromatin state to more transcriptionally active or inactive configurations by introducing transcriptional activators or silencers results in an imbalance of H3K4me2 and H3K9me3 and leads to loss of the centromere (Nakano et al. 2008). However, there seems to be some plasticity, as inducible establishment of H3K9me3 or H3K27me marks at human artificial chromosomes causes a reduction of H3K4me2, but centromere function is maintained (Martins et al. 2016). This (temporary) tolerance for repressive marks may be important to prevent inactivation of centromeres by spreading of silent chromatin states from the pericentromere to the centromere core. One of the main contributors identified so far to antagonize spreading of heterochromatin to the centromere core is the histone acetyltransferase KAT7/HBO1/MYST2 (Ohzeki et al. 2016). It is associated with Mis18—part of the CENP-A loading machinery—at the centromere core in G1 phase and promotes nucleosome turnover, thus preventing accumulation of H3K9me3 and centromere inactivation. A finely tuned chromatin landscape is presumably required to provide the required stability and three-dimensional arrangement of centromeric chromatin during mitosis.

The importance of histone modifications associated with transcriptionally active chromatin indicated that at least some regions of the centromere are actively transcribed. Indeed, centromeric RNA polymerase II (RNA Pol II) transcripts have been reported in a variety of organisms including humans, rice, *S. pombe*, maize, and *Drosophila*. In many cases, inhibition of transcription leads to loss of centromere function (Chan et al. 2012a,b; Choi et al. 2011; Lam et al. 2006; Quénet and Dalal 2012; Rošić et al. 2014; Sadeghi et al. 2014; Saffery et al. 2003; Topp et al. 2004; Yan et al. 2006; Quénet and Dalal 2014). These studies thus uncovered an essential function of transcription for the integrity of centromeres. Centromere function is relatively tolerant to changes in levels of transcription. Chromatin with high levels of histone H3 acetylated on lysine 9 (H3K9ac) and 10-fold elevation in transcript levels had no substantial effect on kinetochore assembly or function. However, there seems to be an upper limit to the levels that are tolerated, as an increase by ∼150-fold rapidly inactivated centromere function and caused loss of CENP-A (Bergmann et al. 2012). In the tammar wallaby, hypermorphic expression of centromeric small RNAs results in disruption of CENP-A localization (Carone et al. 2013). Whether the centromere defects are a direct consequence of increased transcriptional activity or are caused by the over-abundance of centromeric transcripts remains an open question.

The fact that the DNA sequence underlying centromeres is neither necessary nor sufficient for centromere function makes it unlikely that transcripts fulfill specific sequence requirements. Indeed, transcription is also important for the function of human neocentromeres (Chueh et al. 2009). The contributions of the centromeric RNA transcripts and the process of transcription itself to the formation and the maintenance of centromeres have been difficult to clearly separate. Centromeric transcripts have been shown to be required for CENP-A loading in humans, as depletion of these transcripts leads to mitotic defects (Quénet and Dalal 2014). RNA Pol II is enriched on central domain DNA in *S. pombe*, but only relatively low levels of transcripts are detected, consistent with RNA Pol II stalling during transcription of centromeric DNA (Catania et al. 2015). This stalling may enable chromatin remodeling and the deposition of CENP-A nucleosomes. The chromatin remodeler FACT plays an important role in the incorporation of CENP-A (Okada et al. 2009). Mutation of FACT impairs the maintenance of H3 chromatin on transcribed regions and promotes widespread CENP-A incorporation at non-centromeric sites in *S. pombe* (Choi et al. 2012). FACT interacts with the CENP-A histone chaperone CAL-1 in *Drosophila* and is important for transcription and CENP-A incorporation at the centromere (Chen et al. 2015). Moreover, FACT interacts directly with the CCAN components CENP-T-W and is required for their incorporation at centromeres (Prendergast et al. 2016). In addition to the importance of centromere transcription and centromeric transcripts, processing of centromeric RNA, but not translation, has also been shown to play a role in centromere function (Grenfell et al. 2016).

The presence of chromatin marks associated with open chromatin and transcription likely counteracts the more compacted chromatin found at the pericentric regions and creates a permissive environment for CENP-A incorporation and kinetochore assembly. The precise role of centromeric transcripts in these protein complexes is currently unknown. While it is possible that they simply serve a structural role, it is also possible that they function in the epigenetic maintenance of centromeres via sequence complementarity to centromeric DNA.

### Structure of the centromere core

The centromere in animal cells displays a layered architecture with a central core of pericentric heterochromatin and the two CENP-A-containing chromatin domains of sister chromatids peripherally attached, onto which the kinetochores build to engage with spindle microtubules (Vagnarelli and Earnshaw 2001; McIntosh et al. 2002; Guenatri et al. 2004; Vagnarelli et al. 2008) (Fig. 2b). While the CENP-A domains provide the substrates for attachment of kinetochores, the pericentromeric domain provides elasticity and resistance to tension mediated by cohesin (Gerlich et al. 2006; Ribeiro et al. 2009). The pericentromeric domain is further critical for tension sensing and signaling to the mitotic checkpoint as the chromosome passenger complex including aurora B localizes to the pericentromeric domain. For a detailed review on this topic, see van der Horst and Lens (2014). A well-studied example for this layered architecture is found in mouse, where mouse major satellite repeats are packaged into heterochromatin and form the inner centromere that provides cohesion of sister chromatids, while the minor satellite repeats assemble the CENP-A-containing core centromere and link to the kinetochore (Guenatri et al. 2004).

Quantitative approaches have shown that CENP-A nucleosomes make up only a fraction of core centromeres, arguing that these nucleosomes are interspersed with H3-containing nucleosomes (Bodor et al. 2014). This is also evident from work using three-dimensional deconvolution and super-resolution microscopy of stretched chromatin fibers that revealed alternating blocks of CENP-A-containing and H3-containing nucleosomes in *Drosophila*, chicken, and humans (Blower et al. 2002; Ribeiro et al. 2010). *S. pombe* regional centromeres deviate from this pattern, as their core regions are devoid of H3 nucleosomes (Thakur et al. 2015).

To identify the three-dimensional arrangement of the CENP-A-containing and H3-containing chromatin, a number of groups have analyzed mitotic chromosomes by electron microscopy. CENP-A domains were found to be localized at the surface of the primary constriction and occupy roughly two thirds of the constriction length, one third of the constriction height, and one third of the constriction width, both at human alphoid and neocentromeres (Marshall et al. 2008). A similar pattern is found at barley, wheat, and spelt chromosomes, where CENP-A is restricted to the surface of the primary constriction (Wanner et al. 2015). Interestingly, Wanner et al. also reported that microtubules do attach not only to the primary constriction but also to the chromosomal areas flanking it. In the holocentric plant *Luzula elegans*, metaphase chromosomes form a groove of chromatin to which CENP-A almost exclusively localizes (Heckmann et al. 2011; Wanner et al. 2015; Schubert et al. 2016). In pea chromosomes, the primary constrictions are unusually elongated, exhibit three to five explicit CENP-A-containing regions, and can thus be seen as an intergrade between regional and holocentromeres. Correlative fluorescence and scanning electron microscopy showed that the CENP-A within these domains is also oriented towards the surface, as is seen in organisms with smaller primary constrictions, but interrupted by areas without visible CENP-A (Neumann et al. 2012).

In summary, these studies confirm that CENP-A-containing chromatin occupies only a limited fraction of the centromeric chromatin and reveal that the CENP-A-containing chromatin is mainly exposed towards the surface of the chromosome. How chromatin is folded so that chromosomes form a constriction and that CENP-A nucleosomes are exposed on the surface in a back-to-back orientation during mitosis remains subject to research. Several models have been proposed (Fig. 2c): The chromatin could be coiled into cylindrical structures (solenoid model) or organized in parallel loops (looping model), exposing CENP-A to the outside (Blower et al. 2002). Alternatively, the chromatin could be folded as a layered boustrophedon, with planar sinusoids containing interspersed CENP-A-rich and H3-rich subdomains oriented towards the outer kinetochore (Ribeiro et al. 2010). The coiled centromeric chromatin may be folded or looped back upon itself multiple times in the length dimension to form a multilayered structure (Marshall et al. 2008). Regardless of which model turns out to be closest to reality, the exposed CENP-A nucleosomes coordinate the CCAN protein network described above, which together with the KNL-1/Mis12 complex/Ndc80 complex (KMN) network of the outer kinetochore forms the platform to which microtubules attach (Fig. 2a). Details on the kinetochore structures are described in recent reviews (Pesenti et al. 2016; Nagpal and Fukagawa 2016).

Imaging higher order chromatin to derive folding patterns seems currently beyond the limit of microscopy. Chromosome capture methods could alternatively be used to probe centromeric chromatin of mitotic chromosomes (Naumova et al. 2013). However, this approach will only be feasible once the sequences at satellite centromeres are better assembled, so that interactions can be mapped to the assembly.

### Characterization of pericentromeric chromatin

Centromeres can be divided into centromere core and pericentromeric regions, where the core regions provide the attachment site for the kinetochore and the pericentromere is responsible for sister chromatid cohesion. The major task of centromeres is the capture of microtubules from both spindle poles and to establish bi-orientation of chromatids during mitosis or of bivalents during meiosis. Once attached, centromeres provide the load-bearing point for the spindle forces that act on chromosomes. The mechanical resistance is provided by pericentric chromatin and the ring-like molecules cohesin and condensin that act as tethers for chromatin loops. The tension generated by proper bi-orientation serves as a signal for the spindle assembly checkpoint for control of the cell cycle. Once mitosis progresses to anaphase, the mechanical resistance is rapidly dissipated by proteolytic cleavage of cohesin through the protease separase, thus releasing chromatids to migrate to separate poles. Here, we will review the current knowledge of the chromatin structure at the pericentromere and its relationship to cohesin and condensin.

### Pericentromeric chromatin stabilizes cohesin

**Fig. 3:**
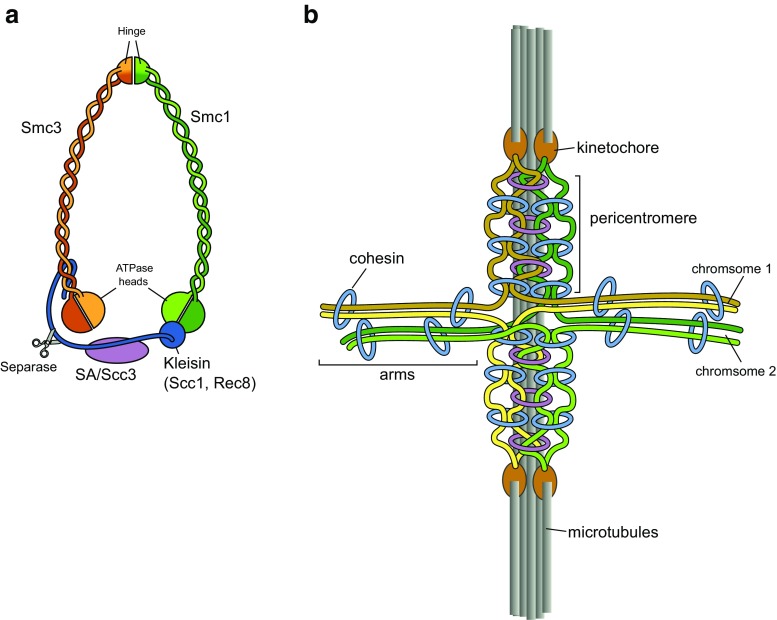
Cohesin shapes the centromere. **a** Model of the *S. cerevisiae* cohesin molecule. **b** Schematic representation of two *S. cerevisiae* sister chromosomes attached to the mitotic spindle showing how cohesin shapes the centromeres in yeast mitosis. Cohesin connects sister chromatids at chromosome arms but also links together centromeric chromatin, both by intrachromosome (*blue*) and interchromosome (*purple*) strand trapping, thereby forming a barrel-shaped structure underneath the kinetochore

Cohesion of sister chromatids is mediated by the stabilization of the cohesin complex on pericentromeric chromatin (Nasmyth and Haering 2009). Cohesin consists of the two structural maintenance of chromatin (SMC) subunits Smc1 and Smc3, both of them folded back onto themselves to form a long coiled coil with N-terminal and C-terminal ABC-type ATPase domains at one end and the central hinge domain at the other (Fig. 3a). Smc1 and Smc3 heterodimerize through the hinge domains, thereby forming long, V-shaped heterodimers. The open ends of the V are bridged by the kleisin subunit Scc1 and the Scc3 subunit to form a ring that can trap two strands of chromatin (Gruber et al. 2003; Haering et al. 2008). During G1 and S phases, cohesin is mobile and is dynamically loaded by the cohesin loader Scc2/Scc4 and released from chromosomes by Wapl/Rad61 (Chan et al. 2012b; Lopez-Serra et al. 2013). During DNA replication, cohesin becomes stabilized through acetylation of the Smc3 subunit by the Eco1 acetyltransferase, which blocks Wapl from triggering the opening of the cohesin ring (Marston 2014).

While stabilization of cohesin during replication links together the entire arms of the sister chromatids, pericentromeres have additional mechanisms to preferentially enrich cohesin. In *S. cerevisiae*, a 25–50 kb region around the kinetochore attachment point is highly enriched in cohesin (Blat and Kleckner 1999; Megee et al. 1999; Tanaka et al. 1999) and corresponds to the pericentromere that, in contrast to regional centromeres, lacks typical heterochromatin. Scc2/Scc4 interacts with the kinetochore (Fernius and Marston 2009; Ng et al. 2009) and loads cohesin in the core region from where it distributes into the surrounding pericentromere (Hu et al. 2015).

In contrast to *S. cerevisiae*, *S. pombe* has extensive pericentric heterochromatin similar to animal and plant centromeres. The *S. pombe* heterochromatin system is extremely well characterized and relies on silencing by both the chromatin and RNA interference (RNAi) machinery to control transcription, recombination, and structural aspects of chromosomes at centromeres, telomeres, and the silent mating-type loci (Grewal and Jia 2007). The RNAi pathway degrades transcripts in cis, while histone deacetylation and histone H3K9 methylation established by chromatin-modifying machinery result in transcriptional gene silencing (Alper et al. 2012; Holoch and Moazed 2015). H3K9 is methylated by a single histone methyltransferase, Clr4, which provides a binding site for the heterochromatin protein 1 (HP1) homologs Swi6 and Chp2. Heterochromatin formation is required for cohesin enrichment at *S. pombe* centromeres, as Swi6 provides the platform for cohesin binding (Bernard et al. 2001; Nonaka et al. 2002), and phosphorylation of Swi6 regulates its effect on sister chromatid cohesion (Bailis et al. 2003). The cohesin subunit Psc3 (Scc3 in *S. cerevisiae* and STAG1/2/3 in humans) interacts with Swi6, and localization of Psc3 through fusion of two chromodomains restores cohesion in the absence of Swi6 (Yamagishi et al. 2008).

Cohesin on pericentromeres is protected from turnover by the recruitment of shugoshin proteins, thus ensuring sister chromatid cohesion (Kitajima et al. 2004; Katis et al. 2004; Kiburz et al. 2005). Shugoshin’s protective function is mediated by the recruitment of the PP2A phosphatase that prevents phosphorylation of the Scc1 subunit of cohesin and thereby keeps separase cleavage at a low level until anaphase (Salic et al. 2004; Kitajima et al. 2006; Riedel et al. 2006). While *S. pombe* shugoshin is required exclusively for cohesion of homologous chromosomes in meiosis I, in *S. cerevisiae* and animals, shugoshin plays a critical role in protecting cohesion during mitosis.

Suv39h and HP1 influence the cohesion of the pericentromere, as loss of H3K9me and HP1 binding lead to increased separation of major satellites during metaphase. However, some groups have reported that the HP1-Suv39 axis does not affect the localization of cohesin to the pericentromere (Koch et al. 2008; Serrano et al. 2009), while more recent reports document a significant contribution of the Suv39-HP1 system to cohesin recruitment, mediated by the H4K20 methyltransferase Suv4-20h2 (Shimura et al. 2011; Hahn et al. 2013). In mammalian cells, cohesin recruitment and stabilization at centromeres during mitosis are more complex, likely because shugoshin-based and HP1-based pathways intermingle, and seem to depend on tissue type and developmental stages.

### The structure and function of pericentromeric chromatin in yeasts

The centromere in *S. cerevisiae* has been studied extensively in order to understand the properties of chromatin at the centromere. The 16 chromosomes have well-defined centromeres that are accessible to sequencing methods since they are non-repetitive. Upon bi-orientation during metaphase, the 32 centromeres of the replicated chromosomes cluster into two foci (Goshima and Yanagida 2000) and connect to 16 microtubules from each side (Winey et al. 1995). While the chromosome arms remain together, chromatids need to dissociate in a ∼10 kb region around the centromere core resulting in the separation of the kinetochores by ∼1 μm (Goshima and Yanagida 2000; He et al. 2000; Tanaka et al. 2000). In order to reconcile the separation of sister kinetochores with cohesin enrichment, Bloom and colleagues proposed that in contrast to the intermolecular tethering that holds together chromatids in the chromosome arms, cohesin at the centromere is engaged in intramolecular trapping of pericentromeric chromatin to produce a loop with the kinetochore at its tip (Verdaasdonk and Bloom 2011; Stephens et al. 2011; Stephens et al. 2013), thereby giving the mitotic chromosome a cruciform structure (Fig. 3b).

The kinetochores and the intervening chromatin structure act against the spindle forces in an elastic manner. One component contributing elasticity is chromatin itself (Bouck and Bloom 2007), which cooperates with condensin and cohesin to establish the spring-like bi-oriented kinetochore structure (Stephens et al. 2011). GFP-tagged cohesin observed in live cells during mitosis was found to form a barrel that extends between the kinetochores (Yeh et al. 2008). This cohesin barrel overlaps with pericentromeric chromatin and encircles the spindle microtubules. In contrast, condensin is found running down the center of the barrel in close proximity to the spindle microtubules (Stephens et al. 2011).

In *S. pombe*, a picture is emerging where pericentromeric chromatin interacts extensively among arms of the same chromosome and among arms of different chromosomes. Recent data from chromosome conformation capture (Duan et al. 2010; Burton et al. 2014; Mizuguchi et al. 2014) show typical “X” patterns off the diagonal that mark centromere-centromere interactions. This prototypical pattern can be used to infer the centromere locations in yeast species for which centromeres have not been mapped previously (Varoquaux et al. 2015). In *S. pombe*, the proximity of centromeres is only mildly reduced in cohesin mutants but almost completely lost in mutants deficient in pericentric heterochromatin formation (Mizuguchi et al. 2014). This indicates that heterochromatin plays a dominant role in promoting the interaction between centromeres in this system.

Taken together, these data suggest that yeast pericentromeres form an intrachromosomal and interchromosomal meshwork at centromeres that is held together by cohesin and heterochromatin proteins (Fig. 3b). This meshwork is important for the sister chromatid cohesion and the establishment of a tension-resisting elastic structure that is required for proper chromosome segregation.

## Conclusions

The three-dimensional organization of the centromeric chromatin is a critical factor for a functional chromosome segregation machinery. It is based on the specialized structural features of the CENP-A nucleosomes that allow assembly of the kinetochore and is evident in the arrangement of the core centromere and pericentromere domains in the context of segregating chromosomes that present the spindle attachment points and monitor their correct engagement and timely resolution of cohesion. Recent progress has started to elucidate many of the details of how the kinetochore interacts with the CENP-A nucleosome. Chromosome conformation capture and microscopy are revealing the higher order structures that centromeres adopt and show that in yeast, centromeres cluster together in interphase and also during mitosis. How the centromere chromatin is folded in detail to establish a tension-resistant structure and how the spatial arrangement into inner and outer centromeric chromatin is established remain open questions. In this review, we have synthesized the wealth of new insight that has been obtained in recent years, revealing an intricately balanced system of transcription, chromatin variants, and modifications that tightly cooperates with the structural and regulatory machinery of the kinetochore in order to guarantee faithful and robust segregation of the genomic material.
